# Genetic parameters and risk factors for abortion traits in Murciano-Granadina dairy goats: A Bayesian multivariate threshold approach

**DOI:** 10.1016/j.vas.2026.100673

**Published:** 2026-04-24

**Authors:** Mohammad Mohseni Takalu, Masood Asadi Fozi, Morteza Mokhtari

**Affiliations:** a Department of Animal Science, Faculty of Agriculture, Shahid Bahonar University of Kerman, Kerman, Iran; b Department of Animal Science, Faculty of Agriculture, University of Jiroft, Jiroft, Iran

**Keywords:** Bayesian approach, Correlation, Heritability, Logistic regression, Prenatal loss

## Abstract

In this study, pedigree information and 9591 records related to the kidding outcomes of Murciano-Granadina goats were used to investigate the risk factors associated with abortion and estimate genetic parameters for abortion across three parities, including Abortion1, Abortion2, and Abortion3 in the Murciano-Granadina goat breed. Data were collected from 2017 to 2023 in a large commercial dairy herd located in Ghale-Ganj City, Kerman province, Iran. The averages for the incidence rates of abortion in the first, second, and third parity were 15.00%, 18.00%, and 15.00%, respectively. The effects of abortion year and abortion season were statistically significant for all the investigated abortion traits, while the age of the doe at abortion significantly influenced Abortion1 and Abortion2 (p < 0.01), but not Abortion3 (p > 0.05), while warm season (lasts from March to September) and younger does were high-risk for abortion. Genetic analysis was performed by applying a Bayesian multivariate threshold model. Posterior means ± posterior standard deviation (PSD) for the heritability estimates were statistically significant values of 0.25 ± 0.10, 0.11 ± 0.04, and 0.19 ± 0.07 for Abortion1, Abortion2, and Abortion3, respectively. Posterior means ± PSD for genetic correlations among abortion traits ranged from 0.23 ± 0.14 (Abortion1-Abortion2) to 0.48 ± 0.16 (Abortion2-Abortion3), with the genetic correlation between Abortion1 with Abortion2 and Aborion3 being statistically non-significant. Posterior means ± PSD for phenotypic correlations among the abortion traits were generally low, ranging from -0.07 ± 0.05 to 0.14 ± 0.05, denoting weak correlation among traits. The obtained results highlighted the effect of environmental factors such as abortion year, abortion season, and age of abortion alongside low to moderate heritability estimates, suggesting that abortion incidence in the studied population of Murciano-Granadina goats was controlled by both genetic and non-genetic factors. The results provided valuable insights for breeding and management strategies aimed at reducing prenatal losses in this breed.

## Highlights

• The averages for the incidence rates of abortion in the first, second, and third parity were 14.32%, 17.00%, and 14.64%, respectively.
• The abortion year, abortion season, and age at abortion were identified as associated risk factors for abortion in the Miurciano-Granadina goat breed.
• Posterior means ± posterior standard deviation for the heritability estimates were statistically significant values of 0.25 ± 0.10, 0.11 ± 0.04, and 0.19 ± 0.07 for Abortion1, Abortion2, and Abortion3, respectively.
• Posterior means ± posterior standard deviation for genetic correlations among abortion traits ranged from 0.23 ± 0.14 (Abortion1-Abortion2) to 0.48 ± 0.16 (Abortion2-Abortion3), with the correlation between Abortion1 and Abortion2 being statistically non-significant.

## 1 Introduction

Abortion in livestock is defined as the premature expulsion of a fetus before full term, a condition typically observed by farmers (Mee, 2020). In small ruminants, flock productivity is strongly dependent on the number of kids and lambs born; thus, effective control of abortion-causing factors is critical for enhancing production profitability (Alemayehu et al., 2021). This issue shows a major constraint in pregnant ewes and does, resulting in substantial economic losses for farmers, particularly in developing countries (Alemayehu et al., 2021). Furthermore, abortion limits flock performance by reducing the pool of replacement animals for herd sustainability and milk yield, while simultaneously increasing the proportion of unproductive females retained for extended periods (Ali et al., 2019). Abortion in sheep and goat flocks is generally multifactorial, with management practices such as healthcare, feeding strategies, and nutritional support during pregnancy exerting a critical influence on fetal survival. Additionally, production systems, seasonal dynamics, and agroecological conditions play a significant role in shaping the incidence of abortion (Alemayehu et al., 2021).

Abortion in goats can disrupt these benefits, impacting food security and economic stability (Amare et al., 2021). Addressing abortion in goats is essential for improving reproductive performance and ensuring the sustainability of livestock industries, particularly in low- and middle-income countries where goats are a key agricultural resource (Muriuki et al., 2019).

The Murciano-Granadina goat breed is regarded as one of Spain’s most important dairy breeds, with a broad international distribution (Miranda et al., 2019). In 2015, the private sector introduced this breed into southern Iran, a region characterized by tropical climates and challenging environmental conditions. The initiative sought to enhance the productivity of native and nomadic goat populations, which traditionally were kept under low-input, low-output systems. By integrating purebred Murciano-Granadina does and bucks into local herds, either directly or through crossbreeding with indigenous breeds, the program aimed to improve production efficiency and thereby strengthen the livelihoods of rural flock holders. Developing appropriate breeding schemes for genetic evaluation of animals requires accurate estimates of genetic parameters for economically important traits. It enables the selection of superior animals to be the parents of the subsequent generations. There is no information on risk factors associated with abortion, and the genetic and phenotypic parameter estimates for abortion traits in this population of the Murciano-Granadina goat breed. Therefore, the current investigation was performed to investigate the risk factors associated with and genetic analysis of abortion in the first three parities of the Murciano-Granadina goat breed.

## 2 Materials and methods

### 2.1 Data and flock management

In this study, pedigree information and records on kidding performance, together with abortion outcomes of Murciano-Granadina does, were used. Data were collected from 2017 to 2023 in a commercial dairy herd located in Ghale-Ganj City, situated in the southern region of Kerman province, Iran. The flock was managed under an intensive production system. Maiden does were first bred at approximately 11 months of age and had an average live weight of 25 kg. They were grouped separately and exposed to fertile bucks at a ratio of 15 does per buck. Pedigree data from 35,121 Murciano-Granadina goats, derived from 937 sires and 11,328 dams, were included in the analysis. The CFC software (Sargolzaei et al., 2006) was employed to identify pedigree errors and to prepare the dataset for subsequent genetic analyses. The pedigree structure of the studied population is presented in Table 1. Animals with both known parents, one known parent, and both unknown parents were 89.81%, 9.56%, and 0.63% of the total registered animals, respectively, implying an appropriate quality of the pedigree used for genetic analysis. The inbred individuals comprised 7.07% of all individuals, with an average inbreeding coefficient of 4.07% in inbred individuals.

**Table 1:** Pedigree structure of the population of the Murciano-Granadina goat.

Item	Numbers
Individuals in total	35,121
Inbreds in total	2484
Sires in total	937
Dams in total	11,328
Individuals with both parents known	31,543
Individuals with both parents unknown	3358
Individuals with one parent unknown	220
Average inbreeding coefficients in the inbreds (percent)	4.07
Maximum of inbreeding coefficients in the inbreds (percent)	31.25
Minimum of inbreeding coefficients in the inbreds (percent)	0.10

### 2.2 Studied traits

The abortion is considered the premature expulsion of the fetus from the uterus before it can survive independently outside and was defined as a binary variable, 1 for natural kidding and 0 for abortion. In this population of the Murciano-Granadina goat breed, the abortions occurred from 50 days to 138 days after pregnancy and were confirmed by a veterinarian. In the data set used, it was recorded as a binary trait with no etiological distinction between infectious and non-infectious causes of abortion. In the present study, abortion in a specific parity was treated as a unique trait. Therefore, abortion in the first (Abortion1), second (Abortion2), and third (Abortion3) parities were considered. Table 2 provides a summary of the descriptive statistics for the abortion traits investigated in this study.

**Table 2:** Descriptive statistics for the abortion traits in the Murciano-Granadina goat breed.

Traits	No. of records	Mean	S.D.
Abortion1	3974	0.15	0.35
Abortion2	3247	0.18	0.37
Abortion3	2370	0.15	0.35

^a^ Abortion1: Occurrence of abortion in the first parity, Abortion2: Occurrence of abortion in the second parity, Abortion3: Occurrence of abortion in the third parity.

### 2.3 Statistical analysis

#### 2.3.1 Non-genetic effects

The following univariate logistic regression model was used to investigate the effect of the predictor variables on the response variable of the abortion status:

$$\log\left(\pi \right)=\beta_{0}\;+\beta_{1}X_{1}\;+\;\beta_{2}X_{2}+\;\beta_{3}X_{3}$$

where π was the probability of abortion; $\beta_{0}$ was the intercept, parameters of $\beta_{1}$ to $,\beta_{3}$ the logistic regression coefficients (parameter estimates) for the explanatory effects of X_1_ to X_3_ were included in the statistical model. The three explanatory variables (X_1_ to X_3_) were year of abortion, with seven levels (2017 to 2023), season of abortion, with two levels (the first season from March to September and the second from October to February), and age of the doe at abortion, with four levels (1–4 years old). Univariate logistic regression models were used by employing the maximum likelihood method of the LOGISTIC procedure in SAS 9.4 software (SAS, 2010).

#### 2.3.2 Genetic analysis

In the present study, two types of models, including univariate and multivariate models, were considered and evaluated for genetic analysis. Under any of these models, abortion was considered as a threshold trait with two categories and/or as a linear trait by assuming a liability scale for abortion.

##### 2.3.2.1 Univariate model

In this model, the genetic parameter estimation for any of the Abortion1, Abortion2, and Abortion3 traits was carried out using a univariate animal model, which can be described as follows:

y=Xb+Za+e

In the model, **y** denotes the record of abortion status in any of the parities, while **b, a**, and **e** represent the vectors of fixed, additive genetic, and residual effects, respectively. The design matrices **X** and **Z** associate the fixed and additive genetic effects, respectively, with the abortion status records.

##### 2.3.2.2 Multivariate model

In this model, the following multivariate animal model was employed for estimating genetic parameters of the investigated abortion traits:

$$y_{i}=X_{i}\;b_{i}+Z_{i}\;a_{i}+e_{i}$$

In the model, **y**_i_ denotes the record of abortion status in the i^th^ parity, while bi, ai, and ei denote the vectors of fixed, additive genetic, and residual effects, respectively. The design matrices X_i_ and **Z**_i_ associate the fixed and additive genetic effects, respectively, with the abortion status records in the *i*^th^ parity.

Within a Bayesian framework, the additive genetic effect was assigned a prior distribution modeled as multivariate normal with mean zero and variance **A**$\sigma_{a}^{2}$, where **A** is the additive numerator relationship matrix and $\sigma_{a}^{2}$ denotes the additive genetic variance. Residual effects were assumed to follow a multivariate normal distribution with mean zero and variance $I_{n}\sigma_{e}^{2}$, with $I_{n}$ representing an identity matrix of dimension equal to the number of individual records and $\sigma_{e}^{2}$ the residual variance.

Genetic analysis was performed with the GIBBS2F90 and THRGIBBS1F90 software (Misztal et al., 2002) for linear and threshold models, respectively. For this purpose, 200,000 iterations were performed, applying a thinning interval of 100, while the first 20,000 iterations were excluded as burn-in. Consequently, 1800 samples remained for estimating characteristics of the posterior distribution. Posterior means and posterior standard deviations (PSD) of the parameters were subsequently obtained using the POSTGIBBSF90 program (Misztal et al., 2002). Convergence for the MCMC chains was evaluated by visual inspection of trace plots for the posterior samples of parameters. Furthermore, the effective sample size (ESS) was also considered as a diagnostic measure of convergence for MCMC samples (Burkner, 2017).

### 2.4 Model comparison

The investigated traits were genetically analyzed under four models, including linear univariate, threshold univariate, linear multivariate, and threshold multivariate models. The predictive performances of these models were assessed via five-fold cross-validation, in which the dataset was randomly split into training and testing subsets of equal size. Fixed and random effects were estimated from the training subset and used to predict records in the testing subset. Predictive ability was quantified using PREDICTF90 (Misztal et al., 2002) by calculating Pearson’s correlation coefficient between observed and predicted values (r(y,$\hat{y}$)). Correlations were averaged across replicates, with higher values indicating greater accuracy. The model with the highest r(y,$\hat{y}$) was used for genetic analysis and estimation of genetic parameters for the investigated abortion traits.

## 3 Results

### 3.1 General considerations

**Fig. 1:**
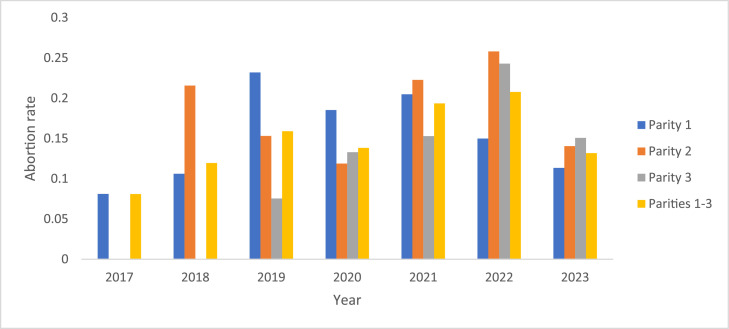
Annual averages for abortion rate in the first, second, third, and as cumulative in the first three parities of the Murciano-Granadina goat breed.

As shown in Table 2, the averages for the incidence rates of abortion in the first, second, and third parity were 15%, 18%, and 15%, respectively. By considering all three parities together, the average for the incidence rate of abortion in the studied population of the Murciano-Granadina goat was 16%. The changes in the abortion rate across the years, for the first, second, third, and cumulative rates in the first three parities, were shown in Fig. 1.

### 3.2 The associated risk factors

Statistically significant testing of the investigated risk factors associated with the considered abortion traits in the Murciano-Granadina goat breed was shown in Table 3. The effects of year of abortion and season of abortion on the investigated abortion traits were statistically significant for all the abortion traits (p < 0.01). Age of the doe at abortion showed statistically significant effects on Abortion1 (p < 0.05) and Abortion2 (p < 0.01), but not on Abortion3 (p > 0.05).

**Table 3:** Risk factors associated with abortion traits in the Murciano-Granadina goat breed.

Variable	Level	Traits a					
		Abortion1		Abortion2		Abortion3	
		Odd Ratio (95% CI)	P-value	Odd Ratio (95% CI)	P-value	Odd Ratio (95% CI)	P-value
Year of abortion	-	-	0.01	-	0.01	-	0.01
	2017	Reference	-	-	-	-	-
	2018	2.48 (1.81–3.41)	-	Reference	-	Reference	-
	2019	2.11 (1.50–2.97)	-	0.89 (0.64–1.23)	-	1.84 (1.05–3.21)	-
	2020	1.98 (1.50–2.62)	-	1.78 (1.25–2.54)	-	2.14 (1.13–4.04)	-
	2021	1.35 (0.98–1.85)	-	2.05 (1.55–2.71)	-	4.03 (2.09–7.76)	-
	2022	1.22 (0.86–1.71)	-	0.93 (0.67–1.30)	-	2.03 (1.07–3.87)	-
	2023	1.43 (0.93–2.19)	-	1.27 (0.88–1.83)		2.54 (1.33–4.83)	-
Season of abortion b	-	-	0.03	-	0.01	-	0.04
	First	Reference	-	Reference	-	Reference	-
	Second	0.81 (0.66–0.99)	-	0.53 (0.22–0.90)	-	0.77 (0.61–0.95)	-
Age of the doe at abortion	-	-	0.01	-	0.01	-	0.06
	1-yr	Reference	-	-	-	-	-
	2-yr	0.43 (0.33–0.56)	-	-	-	-	-
	-	-	-	-	-	-	-
	2-yr	-	-	Reference	-	-	-
	3-yr	-	-	0.59 (0.46–0.76)	-	-	-
	-	-	-	-	-	-	-
	3-yr	-	-	-	-	Reference	-
	4-yr	-	-	-	-	0.73 (0.53–1.01)	-

a Abortion1: Occurrence of abortion in the first parity, Abortion2: Occurrence of abortion in the second parity, Abortion3: Occurrence of abortion in the third parity.

b kidding season with two levels, the first season from March to September and the second from October to February.

### 3.3 Predictive ability of models

The values of Pearson's correlation coefficient between observed and predicted values (r(y,$\hat{y}$)) of abortion traits under different models were shown in Table 4. By considering the abortion status in each parity as a distinct trait, the threshold multivariate model performed better than other investigated models in terms of the highest r(y,$\hat{y}$) for Abortion1 (0.61), Abortion2 (0.53), and Abortion3 (0.51).

**Table 4:** Pearson's correlation coefficient between observed and predicted values (r(y,$\hat{y}$)) under different models.

Trait	Model	r(y,$\hat{y}$)
Abortion1	Linear univariate	0.37 ⁎⁎
	Threshold univariate	0.41 ⁎⁎
	Linear multivariate	0.38 ⁎⁎
	Threshold multivariate	0.61 ⁎⁎
Abortion2	Linear univariate	0.26 ⁎⁎
	Threshold univariate	0.30 ⁎⁎
	Linear multivariate	0.33 ⁎⁎
	Threshold multivariate	0.53 ⁎⁎
Abortion3	Linear univariate	0.24 ⁎⁎
	Threshold univariate	0.28 ⁎⁎
	Linear multivariate	0.37 ⁎⁎
	Threshold multivariate	0.51 ⁎⁎

^a^ Abortion1: Occurrence of abortion in the first parity, Abortion2: Occurrence of abortion in the second parity, Abortion3: Occurrence of abortion in the third parity.

⁎⁎ Significant at p < 0.01.

### 3.4 Genetic parameters

**Fig. 2:**
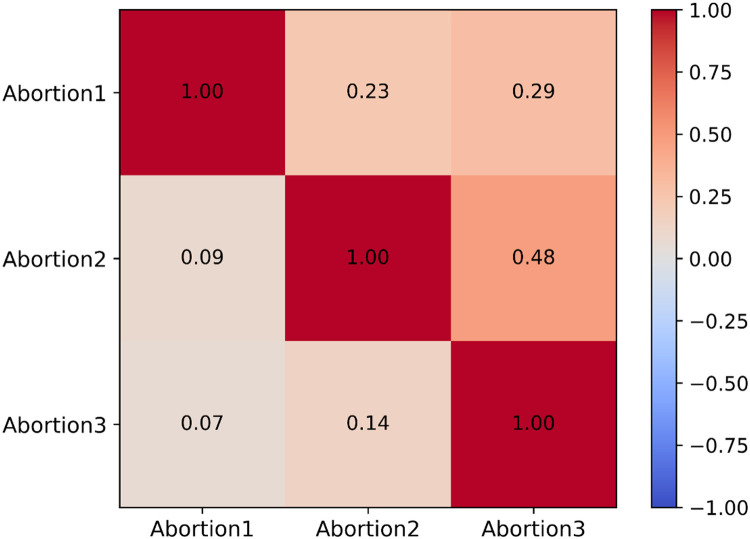
Visual representation of genetic (above diagonal) and phenotypic (below diagonal) correlations among the investigated abortion traits in the Murciano-Granadina goats from multivariate analyses. Abortion1: Occurrence of abortion in the first parity, Abortion2: Occurrence of abortion in the second parity, Abortion3: Occurrence of abortion in the third parity.

As shown in Table 5, posterior means of heritability estimates ± posterior standard deviation (PSD) for abortion in the first, second, and third parities were 0.25 ± 0.10, 0.11 ± 0.04, and 0.19 ± 0.07, respectively, and statistically significant (95% of the highest posterior density or HPD intervals did not include zero). Posterior means ± PSD for genetic and phenotypic correlations among the abortion traits were also presented in Table 4. Genetic correlations for Abortion1-Abortion2, Abortion1-Abortion3, and Abortion2-Abortion3 were 0.23±0.14, 0.29±0.28, and 0.48±0.16, respectively. Posterior means for the genetic correlations of Abortion1 with both Abortion2 and Abortion3 were not statistically significant (95% of HPD intervals included zero). Still, the posterior mean for the genetic correlation between Abortion2 and Abortion3 was statistically significant (95% of HPD intervals did not include zero). The same trends were observed for the phenotypic correlations. Phenotypic correlations for Abortion1-Abortion2, Abortion1-Abortion3, and Abortion2-Abortion3 were 0.09 ± 0.05, −0.07±0.05, and 0.14 ± 0.05, respectively. Fig. 2 showed visual representation of genetic and phenotypic correlations among the investigated abortion traits.

**Table 5:** Posterior means ± posterior standard deviation a for heritability estimates (on diagonal), genetic (above diagonal), and phenotypic (lower diagonal) correlation of abortion traits in the Murciano-Granadina goats from multivariate analyses.

Trait b	Abortion1	Abortion2	Abortion3
Abortion1	0.25±0.10 (0.05:0.45)	0.23±0.14 (−0.04:0.50)	0.29±0.28 (−0.26:0.84)
Abortion2	0.09±0.05 (−0.01:0.19)	0.11±0.04 (0.03:0.19)	0.48±0.16 (0.17:0.79)
Abortion3	0.07±0.05 (−0.03:0.17)	0.14±0.05 (0.04:0.24)	0.19±0.07 (0.05:0.33)

a 95% highest posterior density intervals are shown in parentheses.

b Abortion1: Occurrence of abortion in the first parity, Abortion2: Occurrence of abortion in the second parity, Abortion3: Occurrence of abortion in the third parity.

## 4 Discussion

### 4.1 Incidence rate of abortion across parities

In sheep and goats, abortion was defined as the loss of a fetus before 140 days of pregnancy (Tulu et al., 2020). The abortion rate was determined as a critical factor in assessing the reproductive efficiency of a goat herd, as it directly influenced the number of successful births and overall herd productivity (Toplu & Altinel, 2008). As shown in Fig. 1, in parity 1, the abortion rate increased from about 8% in 2017 to about 23% in 2019, and decreased afterwards to about 11% in 2023. In parity 2, the abortion rate had fluctuations; the abortion rate decreased from 21% in 2018 to 12% in 2020, increased afterwards to 26% in 2022, and then decreased to 14% in 2023. In parity 3, the abortion rate increased from 7% in 2019 to 24% in 2022 and decreased to 13% in 2023. By considering all three parities, the abortion rate generally increased from 8% in 2017 to 24% in 2022 and then decreased to 15 in 2023 (Fig. 1).

Reported abortion rates in goats vary widely among breeds, regions, and production systems. Mishra et al. (2024) studied the abortion rates in three Indian goat breeds, including Barbari, Jakharana, and Jamunapari, which were reared under a semi-intensive production system and reported average abortion rates of 2.27%, 1.16%, and 2.58%, respectively. They also reported an average abortion rate of 1.82% in these breeds, which was lower than the overall abortion rate obtained in the present study (16%). Assefa et al. (2024) reported an overall abortion rate of 29.80% in three goat breeds in Ethiopia, which was higher than the corresponding rates obtained in our study. Mishra et al. (2023) reported the mean annual abortion rate in Indian goats as 9.16% under extensive field conditions. Kebede et al. (2011) reported an overall mean of 3.8% in Arsi-Bale goats. Variations in abortion rates could be due to various factors such as infectious, climatic, and nutritional factors (Rojero et al., 2005). Mee (2023) pointed out that the differences in abortion rates may be attributed to the variations in breed and the management practices performed at the farm level.

### 4.2 Non-genetic effects

For Abortion1, the OR decreased from 2019 to 2023 and increased in 2024. For both Abortion2 and Abortion3, the OR increased from 2020 to 2022, decreased in 2023, and increased in 2024. The significant effect of year of abortion on the abortion rate was possibly influenced by variations in management practices, especially health practices, or environmental conditions over the studied years (Kebede et al., 2012). Darlay et al. (2014) pointed out that the effect of year of abortion on the abortion rate might highlight differences in husbandry practices that can be used to reduce the likelihood of abortion. Significant effect of year on abortion in Arsi-Bale goats reported by Kebede et al. (2012).

For all the traits studied, the OR decreased in the cold season (from October to February). It may be concluded that the incidence rates of abortion increased in the warm season (from March to September), which can be explained by heat stress in the warm season (Yaqoob et al., 2016). Mishra et al. (2023) noted that the occurrence of abortions in goats reared at organized farms under semi-intensive systems and field conditions varied significantly across different seasons (rainy, winter, and summer) (p < 0.01). They reported the highest occurrence of abortion in the winter season, which was not in agreement with that observed in the present study. Kebede et al. (2012) reported that the effect of season on abortion in Arsi-Bale goats was non-significant.

For Abortion1 and Abortion2, older does had a lower OR. In the first parity, 2-year-olds had a lower incidence of abortion than 1-year-old goats, and in the second parity, 3-year-old and older does had a lower incidence of abortion than 2-year-old ones. But for Abortion3, there was no significant difference in the incidence rate of abortion between 3-year-old and 4-year-old does. Significant reduction in the incidence of abortion with the increase in age at the abortion reported in African goats (Mourad, 1994). Assefa et al. (2024) reported significant effects of year, season, and age on abortion in the native breeds of goat in Ethiopia. They noted that the likelihood of goats experiencing abortion during the dry season was higher compared to those in the wet season. They also pointed out that goats that were two years old or younger had a lower OR for experiencing abortion than goats older than two years, which was in contrast with what was observed in the present study.

### 4.3 Model selection for genetic analysis

The predictive performance of four fitted models, including linear univariate, threshold univariate, linear multivariate, and threshold multivariate models, was investigated. The threshold multivariate model performed better than other models. In animal breeding, the main purpose of data analysis is the estimation of the genetic parameters for economically important traits (Luo et al., 2001). Best Linear Unbiased Prediction (BLUP) has been widely applied to estimate genetic parameters for normally distributed traits, because it yields the maximum likelihood estimator of the best linear predictor (Wang et al., 1993). Luo et al. (2001) pointed out that for estimating variance components for categorical traits, Bayesian inference had more advantages than the conventional BLUP method due to the non-normal distribution of these traits. In the present study, abortion was defined as a trait with two categories which the normal distribution does not hold. Therefore, a Bayesian approach had enough justification for the genetic analysis of the investigated traits.

### 4.4 Estimates of heritability, genetic and phenotypic correlations

In the present study, abortions were defined with no etiological distinction between infectious and non-infectious factors associated with this trait. Therefore, estimated values of genetic parameters for these traits may be interpreted with caution. The heritability estimates for the investigated abortion traits were low to medium, ranging from 0.11 for Abortion2 to 0.25 for Abortion1, indicating that additive genetic variation had lower contributions in the expression of Abortion2 than other investigated traits. It may be concluded that additive genetic effects had more influence on abortion occurrence in the first parity of the studied population of the Murciano-Granadina goat breed than second and third parties.

However, the reduction in heritability during the second parity (0.11) followed by an increase in the third (0.19) may partly reflect the greater contributions of non-additive genetic effects or environmental factors such as variations in management, disease exposure, or physiological adaptation of does across the later parities. To our knowledge, there were no published estimates on the heritability of abortion in the goat breeds in distinct parities. Therefore, comparison of the heritability estimates obtained for abortion traits in the present study with those reported on other livestock breeds may be justified. Darlay et al. (2014) reported the heritability of abortion in Charollais sheep flocks as 0.08. Nadri et al. (2021) reported a heritability estimate of 0.165 for abortion in Iranian Holstein cows. Amin et al. (2000) reported heritability estimates of 0.12, 0.15, and 0.28 for abortion within 60 days in first, second, and third parities in a Hungarian Holstein population, respectively.

The estimates for the genetic correlations of Abortion1 with both Abortion2 and Abortion3 were positive but not statistically significant. In general, the existence of non-statistically significant genetic correlation between two traits implied different genes involved in the expression of them. But in the present study, as we dealt with the same trait in different parities, such non-significant genetic correlations may be justified partly by high values of PSD for both estimates of genetic correlations between Abortion1 with Abortion2 (0.23±0.14) and Abortion3 (0.29±0.28), mainly originating from small sample size of data. Medium in magnitude, positive, and statistically significant genetic correlation was found between Abortion2 and Abortion3, implying some common genetic mechanisms involved in controlling both traits. The same trends were found for the phenotypic correlations, with low-magnitude phenotypic correlations between Abortion2 and Abortion3.

## 5 Conclusion

The non-genetic and genetic factors influencing abortion in the Murciano-Granadina goat breed under intensive production systems were examined. Results indicated that additive genetic variations for abortion traits were relatively low. Therefore, direct genetic improvement in abortion traits may not be justified. Consequently, it can be inferred that incorporating non-additive genetic effects for enhancing abortion resistance, along with improving environmental and management conditions, would be more crucial when designing a breeding program aimed at lowering abortion rates in the studied herd of Murciano-Granadina goat.

## Funding

This research did not receive any specific grant from funding agencies in the public, commercial, or not-for-profit sectors.

## Data availability

The data that support the findings of this study are available from the corresponding author upon reasonable request.

## Ethical approval

This study did not involve any experimental procedures on live animals. All data used were derived from routinely collected performance and pedigree records from a commercial goat breeding farm.

## Declaration of generative AI use

We used CoPilot to proofread our manuscript before submitting and to correct any inaccurate grammar and punctuation.

## CRediT authorship contribution statement

**Mohammad Mohseni Takalu:** Writing – original draft, Methodology, Formal analysis, Data curation. **Masood Asadi Fozi:** Writing – original draft, Supervision, Methodology, Investigation, Conceptualization. **Morteza Mokhtari:** Writing – original draft, Methodology, Formal analysis, Data curation, Conceptualization.

## Declaration of competing interest

The authors declare that they have no known competing financial interests or personal relationships that could have appeared to influence the work reported in this paper.
